# Sodium Alginate/Gelatine Hydrogels for Direct Bioprinting—The Effect of Composition Selection and Applied Solvents on the Bioink Properties

**DOI:** 10.3390/ma12172669

**Published:** 2019-08-22

**Authors:** Dorota Bociaga, Mateusz Bartniak, Jacek Grabarczyk, Karolina Przybyszewska

**Affiliations:** Faculty of Mechanical Engineering, Institute of Materials Science and Engineering, Lodz University of Technology, 1/15 Stefanowskiego St., 90-924 Lodz, Poland

**Keywords:** bioprinting, bioink for scaffolds, micro-extrusion, mechanical properties, cell viability, degradation of hydrogels

## Abstract

Hydrogels tested and evaluated in this study were developed for the possibility of their use as the bioinks for 3D direct bioprinting. Procedures for preparation and sterilization of hydrogels and the speed of the bioprinting were developed. Sodium alginate gelatine hydrogels were characterized in terms of printability, mechanical, and biological properties (viability, proliferation ability, biocompatibility). A hydrogel with the best properties was selected to carry out direct bioprinting tests in order to determine the parameters of the bioink, adapted to print with use of the designed and constructed bioprinter and provide the best conditions for cell growth. The obtained results showed the ability to control mechanical properties, biological response, and degradation rate of hydrogels through the use of various solvents. The use of a dedicated culture medium as a solvent for the preparation of a bioink, containing the predicted cell line, increases the proliferation of these cells. Modification of the percentage of individual components of the hydrogel gives the possibility of a controlled degradation process, which, in the case of printing of temporary medical devices, is a very important parameter for the hydrogels’ usage possibility—both in terms of tissue engineering and printing of tissue elements replacement, implants, and organs.

## 1. Introduction

Hydrogels may be defined as a three-dimensional network polymers (synthetic or natural) which have a great ability to absorb large amounts of water or biological fluids, what gives them a high degree of flexibility [1,2,3,4]. Due to their high water content, porosity, and physical state, they simulate natural living tissues better than other synthetic biomaterials [5]. Hydrogel structure is formed by hydration of the hydrophilic groups (amide, amino, carboxyl, and hydroxyl) contained in the polymer network [6]. Description of the hydrogels as the network is determined by the presence of crosslinks, which prevents the breakdown of polymer chains [2]. The properties of hydrogels (including functionality, reversibility, biocompatibility) meet material as well as biological requirements, so they can be used for treatment, substitution, or interaction with living tissues or organs [1]. What is more, the modification of the physical properties of these biomaterials (such as swelling, surface characteristics, mechanical properties) is possible as a result of physicochemical reactions [6].

Hydrogels are not a new discovery—they have already existed on Earth in the early stages of human life (e.g., biofilms, hydrated extracellular matrix, gelatine, or agar) [1]. The wide spectrum of hydrogel properties and the possibility of modification depending on their application currently resulted in use primarily for the production of hydrogel dressings [7,8,9] and lenses [10,11]. The unique properties of hydrogels similar to the natural tissues and the appropriate structure for cell growth and survival allow for their use in tissue engineering. In addition, due to the ability to control the shape, porosity, and size of scaffolds, hydrogels have found a wide application in intensively developed bioprinting [12,13].

Bioprinting can be defined as the simultaneous placement of biomaterial and living cells layer by layer in a desired pattern to produce living tissues and organs [14,15,16,17]. The advantage of bioprinting over traditional scaffold production methods is the ability to print complex geometries with controlled porosity. In addition, cells (as well as other biological components) can be introduced into the hydrogel which can be precisely formed in a desired pattern [18]. There are many methods of bioprinting [19], among others: material extrusion (pneumatic/mechanical, hybrid printing) [18,20], material jetting (piezoelectric/thermal inkjet, acoustic wave jetting, electrohydrodynamic jetting, and laser-induced forward transfer) [21] and vat polymerization (stereolithography, digital light projection, and two-photon polymerization) [22]. The most widely-used and most developed method is the micro-extrusion method due to the low cost, good integrity between 3D-printed layers and very high cell viability (even more than 98%) [18,22,23]. Micro-extrusion method was selected in conducted research over other printing techniques. Material extrusion bioprinting, compared to the material jetting method, allows one to use a wide range of materials (with possibility to extrude low and high viscosity material). This is due to the fact that nozzles with larger diameters are used with the possibility of modifying their shapes. What is more, it is possible to bioprint homogeneous and heterogeneous structures with good uniformity of cell’s distribution within 3D printing structures [18,22,24,25]. Many hydrogels have already been adapted to printing by micro-extrusion [26,27,28] and one of the most commonly used in bioprinting is blend of sodium alginate with gelatine, which have a good printability and biological properties at the same time as well as fast crosslinking rate [29,30,31]. 

Gelatine is a fibrous protein obtained as a result of partial hydrolysis of the triple helix structure of collagen [32]. What is the most important, gelatine has a high ability to adhere cells due to the presence of RGD domains in the chemical structure [33], but on the other hand it has low mechanical properties without modification (e.g., compressive strength). It is not immunogenic and biocompatible material with high capability to absorb water [31]. In the case of applications of gelatine in 3D bioprinting, the most important is to improve mechanical strength and thus the stability of the printed structure. One of the most effective methods is physical blending with other hydrogels (for example with methacrylamide or sodium alginate) [31,34,35] and chemical or biochemical crosslinking [31,36,37]. Alginate is a polysaccharide, produced from brown algae and bacteria. It is a natural copolymer composed of repeating units of two mers: β-d-mannuronic acid (M) and α-l-guluronic acid (L) [38]. Sodium alginate provides high extrusion capacity, thus enabling the processes of bioprinting, and fast and irreversible crosslinking ensures the stability of the printed structures. It is a biocompatible material. It is characterized by limited ability to adhere cells, but at the same time maintains their viability [39].

Many teams evaluate the usage of sodium alginate/gelatine blends. Pan’s research team [40] in their research on the use of sodium alginate and gelatine in bioprinting, showed that the printed fibers are not smooth along the entire length, and additional chemical crosslinking of gelatine—using glutaraldehyde—limits the rate of degradation of the obtained printout. Research conducted by the Zhang et al. [41] showed a high survival rate of cells suspended in sodium alginate/gelatine hydrogels (although its level decreases with the prolonged incubation time) and the cells are capable to migrate. The hydrogels blends can also be modified—e.g., to obtain methacrylated gelatine (GelMA) crosslinking by UV light. Research on such hydrogels was carried out by the Colosi et al. [42]. In their research, it was shown that crosslinking GelMA with low-frequency UV light below 30 s does not affect the survival of the cells. In addition, mentioned research showed that the cell proliferation and the ability to migrate is limited in rigid hydrogels crosslinked to a high degree. In terms of hydrogels’ applications in bioprinting, sodium alginate/gelatine are also modified by the introduction of growth factors to increase the ability of the cells to proliferate, which was carried out by the Neufurth research team [43]. The obtained results showed high cell survival, indicating that stabilization of sodium alginate with gelatine improves cells’ survival. Giuseppe et al. [44], in their research, showed no negative impact of calcium ion on the cell survival (from the crosslinking process), and the extended crosslinking time increases the strength parameters of the obtained hydrogel.

Many studies have been carried out using sodium alginate/gelatine blend as a hydrogel for EBB bioprinting. In most cases physiological saline [43], deionized water [40], HEPES medium [42], or various concentrations of PBS buffer [45] are used as the solvents. Results proved that type of solvent influence the hydrogels extrusion capability. Nevertheless, there are no studies for solvents, which are culture media dedicated to a given cell line. Investigations of such novel hydrogel (containing the bioink) are significant for bioprinting techniques development. In this work, we have investigated the influence of various solvents and concentration of gelatine onto the mechanical and biological properties as well as hydrogels’ extrusion ability. We have also checked the impact of printing process on cells’ survival after extrusion. Nevertheless, there are no studies for solvents, which are culture media dedicated to a given cell line. Investigations of such novel hydrogel (containing the bioink) are significant for bioprinting techniques development. In this work, we have investigated the influence of various solvents and concentration of gelatine onto the mechanical and biological properties as well as hydrogels’ extrusion ability. We have also checked the impact of printing process on cells’ survival after extrusion. 

## 2. Materials and Methods

### 2.1. Hydrogel Sample Preparation

Two solvents were used for the preparation of hydrogels: deionized water (conductivity of 0.07 μS) and Dulbecco’s modified Eagle’s medium (DMEM, Corning Incorporated, Corning, NY, USA) supplemented with 10% fetal bovine serum (FBS) (Corning Incorporated, Corning, NY, USA) and 1% antibiotic penicillin/streptomycin (P/S) (Corning Incorporated, Corning, NY, USA). This composition corresponds to the complete culture medium that is applied for the cell line applied in this experiment. Table 1 presents the signatures of the sample materials and the content of individual components.

In order to obtain sterile biological material for cell culture, powders of sodium alginate, gelatine, and calcium chloride were subjected to UV radiation for 60 min. First, the sodium alginate (Sigma-Aldrich, Saint Louis, MO, USA) was dissolved in solvent heated to 50 °C using a magnetic stirrer (Bionovo, Legnica, Poland). Gelatine (Sigma-Aldrich, Saint Louis, MO, USA) was added to the dissolved alginate and mixed until complete dissolution. The entire process was carried out under sterile conditions in a laminar chamber (Biogenet, Józefów, Poland). Then the degassing process was conducted (10,000 rpm, 2 min) to eliminate air bubbles and after that, the solution was poured into a beaker. A 2% solution of calcium chloride (Sigma-Aldrich, Saint Louis, MO, USA) was prepared under sterile conditions by dissolving the calcium chloride powder in deionized water (conductivity of 0.07 μS). After mixing, the hydrogels were crosslinked (at the same time for for each tested hydrogel composition). Prepared chloride solution was poured onto the surface of the gels and left for complete crosslinking. All operations were carried out under sterile conditions. Samples were cut out and washed for 30 min in phosphate buffered saline buffer (PBS) (Corning Incorporated, Corning, NY, USA).

### 2.2. Methods

In order to determine the influence of different solvents and gelatine concentration onto the mechanical properties of hydrogels, their biodegradability and extrusion ability, the different evaluation methods were applied.

#### 2.2.1. Viscosity Measurement—Rheometer

The hydrogels were tested in terms of rheological properties using a RheolabQC rheometer (Anton Paar GmbH, Graz, Austria) to determine the curve of the viscosity versus shear rate. The tests were carried out at room temperature, maintaining the temperature of the hydrogels at 20 °C in time of 160 s (in the range of shear stress from 0 to 160 s^−1^). Number of samples to ensure accuracy of the rheological data was three for each tested hydrogel composition. For the hydrogel chosen for the bioprinting, an additional study of temperature influence on its viscosity was carried out. For this purpose, the measuring system (including the hydrogel itself) was heated to 34, 37, and 40 °C. The study aimed to verify the effect of the temperature of the printing nozzle on the viability of the cells contained in the hydrogel.

#### 2.2.2. Mechanical Parameters Measurement—Tribotester

The hydrogels were subjected to a mechanical parameter measurement (uniaxial compression tests of hydrogels). For each material, three samples with a diameter of 14 mm and a height of 9 mm were prepared, and the average value was calculated from the obtained results. The test was carried out until reaching a maximum strain of 70% with a compression speed of 1 mm/s. On the basis of stress strain curves obtained, compressive strength was determined. The tests were carried out on a tribotester (UMT-2, Bruker, Billerica, MA, USA). 

#### 2.2.3. Measurement of Hydrogels Properties after the Degradation Test

Degradation rate is another important property of hydrogels used in tissue engineering. The ideal hydrogel should degrade in vivo at a given time and at a controlled rate to create space for new tissue growth. Prepared cylindrical samples with a diameter of 14 mm and a height of 9 mm were subjected to stability analysis over time. Samples were placed in 12-well plates and 3 mL of PBS buffer was added. The plates were placed in a thermal cabinet at a constant temperature of 37 °C and incubated for 24 h, 48 h, 7 days, and 14 days (separate set of samples for each time).

##### Degradation Rate of Hydrogels

Samples of the tested hydrogels after specific incubation periods in PBS buffer at 37 °C were weighed to determine the degradation rate. For each sample, its starting weight was determined and then weighed after a specified time. On the basis of the results obtained, the degradation rate was calculated according to the formula (1),

(1) $$R=\frac{W_{t}-W_{0}}{W_{0}}*100\%$$

where: R—degradation rate [%], $W_{0}$—starting weight of sample [g], $W_{t}$—weight of sample after specified time [g].

##### Mechanical Properties of Hydrogels

For each incubation period, the samples were subjected to a compressive strength test by performing the uniaxial compression test described in Section 2.2.2. 

#### 2.2.4. Fatigue Measurement—Tribotester

Hydrogels were subjected to fatigue tests using a tribotester (UMT-2, Bruker, Billerica, MA, USA). Hydrogels in the form of cylinder with a diameter of 14 mm and a height of 9 mm were deformed in the elastic range at cycles of 10, 20, and 50. Then the same samples were subjected to uniaxial compression tests to determine the compression strength (three replicates were made for each sample).

#### 2.2.5. Swelling Ratio

According to the procedure commonly described in the literature [40,46], a study was carried out to determine the swelling capacity of the material. The crosslinked hydrogels were allowed to dry in a thermal cabinet at 37 °C for 48 h. The dried samples were weighed, and then 3 mL PBS buffer was added and allowed to stand in a heat cabinet (37 °C). After 1, 3, 6, 24, and 27 h, the samples were weighed again to determine the weight of absorbed water. The swelling ratio was determined according to the formula 1.

#### 2.2.6. Cells Viability/Cytotoxicity Test after Direct Contact with Hydrogels Sample

For testing cells viability in direct contact, samples of 22 mm in diameter and 5 mm in height were prepared. After washing in PBS, hydrogels rings were placed on the culture wells and fixed to the bottom with carbon tape to prevent samples floating. Then, endothelial cells (EA.hy926-ATCC^®^ CRL-2922™, LGC Standards, London, UK) were seeded on the sample at the density of 6 × 10^4^ cells per well and supplemented with 2 mL of full culture medium. For each type of hydrogel, two culture plates were prepared with three replicates of each (cells with full culture medium as a control sample only in one plate), for incubation at 37 °C in a 5% CO_2_ atmosphere and 90% humidity for 24 and 48 h respectively. After the incubation period, the samples were stained with solutions of two dyes prepared in PBS buffer: calcein and ethidium homodimer (Biotium Inc., Fremont, CA, USA).

#### 2.2.7. Cells Viability/Cytotoxicity after Contact with Extracts Made of Hydrogels Sample

Extracts were prepared in accordance with the guidelines of the PN-EN ISO 10993-5. Samples with an area of 6 cm^2^ were placed on the culture wells and supplemented with 2 mL of full culture medium, followed by incubation for 24 h at 37 °C. Endothelial cells (EA.hy926-ATCC^®^ CRL-2922™, LGC Standards, London, UK) in an amount of 6 × 10^4^ cells per well were seeded on the culture plate and supplemented with 2 mL full culture medium. Subsequently, they were incubated for 24 h at 37 °C in a 5% CO_2_ atmosphere and 90% humidity. After this time, the medium was removed and the cells were washed with PBS buffer. Then, the previously prepared extracts in amount of 2 mL per well were added (cells with full culture medium as a control sample only in one plate). Incubation was carried out for 24 and 48 h at 37 °C in an atmosphere of 5% CO_2_ and humidity of 90%. After the incubation period samples were stained with solutions of two dyes prepared in PBS buffer: calcein and ethidium homodimer (Biotium Inc., Fremont, CA, USA). 

#### 2.2.8. Extrusion Ability and Cells Survival Test after Extrusion

Based on the results of previously described tests, hydrogel providing the best cell viability with good mechanical properties was selected to prepare bioink. Powders of sodium alginate, gelatine, and calcium chloride were sterilized using UV radiation for 60 min. The hydrogels were then prepared according to the procedure used to develop the selected hydrogels in a system heated to 37 °C. The suspension of endothelial cells (EA.hy926-ATCC^®^ CRL-2922™, LGC Standards, London, UK) in the culture medium was centrifuged for 5 min at 180 g. The supernatant was collected and the cell pellet was introduced to the hydrogel solution and gently mixed to get uniform cells distribution. The process of the bioprinting was carried out on an individually designed and constructed bioprinter of which motion kinematics is based on the CoreXY printer system printing in FDM technology. The hydrogel was extruded through a flat-tipped needle with a diameter of 430 μm and a length of 16 mm heated to the temperatures: 34, 37, and 40 °C. One-layer strands with a length of 1 mm and a height of 0.35 mm were extruded. The viability test in printouts obtained was evaluated via the live/dead tests.

#### 2.2.9. Statistical Analysis

The quantitative results of the hydrogels were subjected to statistical analysis using a one-way analysis of variance (ANOVA). The results were compared and considered statistically significant if *p* < 0.05. Statistical significance, if present, has been marked on the charts with an asterisk (* for *p* < 0.05 and ** for *p* < 0.01) above the individual results.

## 3. Results

### 3.1. Results of Viscosity Measurement

**Figure 1:**
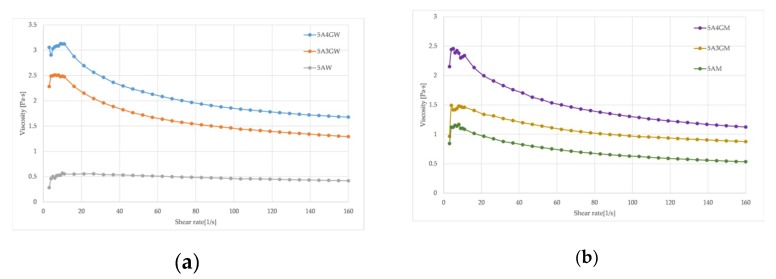
Rheological properties of the samples dissolved in water (W) (**a**) and full culture medium (M) (**b**) with different contents of gelatine.

The possibility of extruding the hydrogel through the nozzle depends on its viscosity—the higher the viscosity of the hydrogel, the higher the pressure necessary to extrude it. High pressure affects the formation of high shear stresses, which in the case of a direct bioprinting will affect cell viability. Figure 1 presents curves of viscosity dependence on shear rate for hydrogels prepared using various solvents.

The introduction of gelatine into the alginate hydrogel increases the viscosity in both solvents. In the case of water, the introduction of 3% *w*/*v* of gelatine increases the viscosity by about 3 times, and with the addition of 4% *w*/*v* the viscosity increases almost 4 times. With the full culture medium this trend is less marked—the introduction of 3% *w*/*v* gelatine increases the viscosity by 0.5 times, and 4% *w*/*v* by about 1.5 times. Dissolving the alginate alone in the medium allows for a higher viscosity than in the case of dissolution in water (5AW and 5AM samples in Figure 1). The highest viscosity is characterized by a hydrogel composed of 5% *w*/*v* of sodium alginate and 4% *w*/*v* of gelatine prepared using water as a solvent. The results of the conducted study confirm that the prepared hydrogels are non-Newtonian fluids (viscosity of the fluid is not constant) and are characterized by the ability of shear thinning. Along with the increase of the shear rate, the viscosity of the hydrogels decreases. 

### 3.2. Results of Mechanical Parameters Measurement

**Figure 2:**
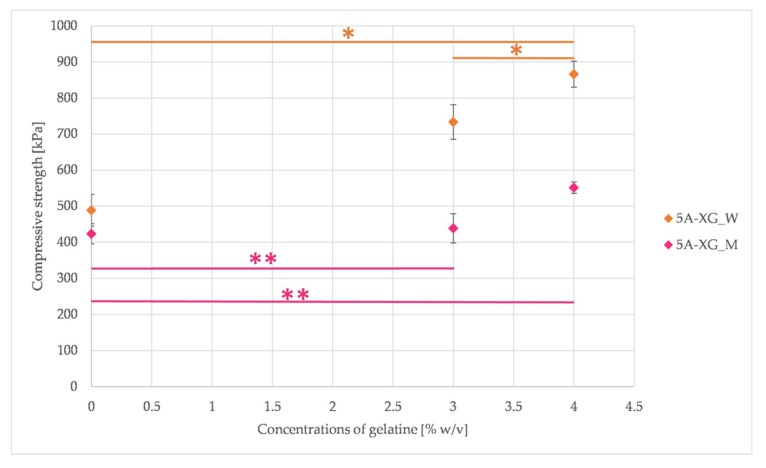
Compressive strength results for the samples dissolved in water (W) and full culture medium (M) with different content of gelatine (* = *p* < 0.05 and ** = *p* < 0.01, results statistically significant).

The gelatine concentration and type of solvent used affects the mechanical properties of the tested hydrogels, as shown in Figure 2.

The compressive strength achieves higher values when using deionized water as the solvent. The difference between the compressive strength of samples without gelatine (5AW and 5AM) is 66 kPa, and its addition makes the difference increase. In the case of both solvents, there is a tendency to increase the compressive strength of hydrogels with the increasing content of gelatine (however, it has a stronger character for water).

### 3.3. Results of Hydrogels Properties Measurement after the Degradation Test

Hydrogels were subjected to a degradation test in the work divided into time intervals of 24 h, 48 h, 7 days, and 14 days, and the results obtained on this basis are presented below. Parameters as degradation rate and mechanical properties were evaluated for the samples after specific periods of degradation.

#### 3.3.1. Results of Hydrogels Degradation Rate of Hydrogels

**Figure 3:**
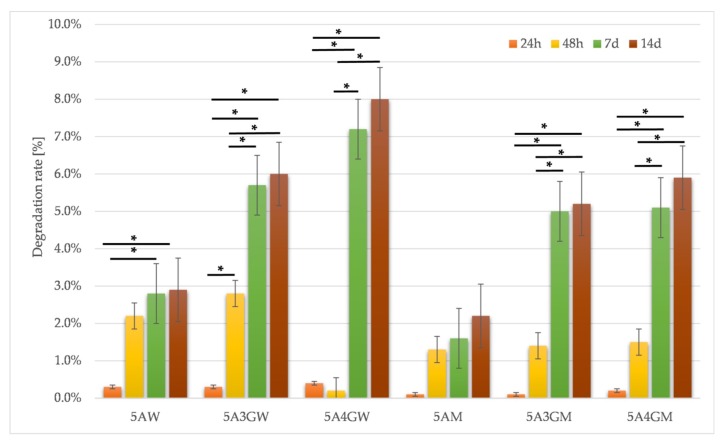
Degradation rates for samples dissolved in water (W) and full culture medium (M) with different content of gelatine evaluated after 24 h, 48 h, 7 days, and 14 days of incubation (* = *p* < 0.05, results statistically significant).

The results of sample degradation rate shown in Figure 3.

On the basis of the results obtained, it can be noticed that greater weight loss occurred in the case of hydrogels prepared using deionized water as a solvent (however, despite a 14-day incubation, the degradation index does not exceed 8%). For both solvents, degradation is accelerated by the addition of gelatine—as the concentration increases, the degradation rate increases as well.

#### 3.3.2. Results of Mechanical Properties

**Figure 4:**
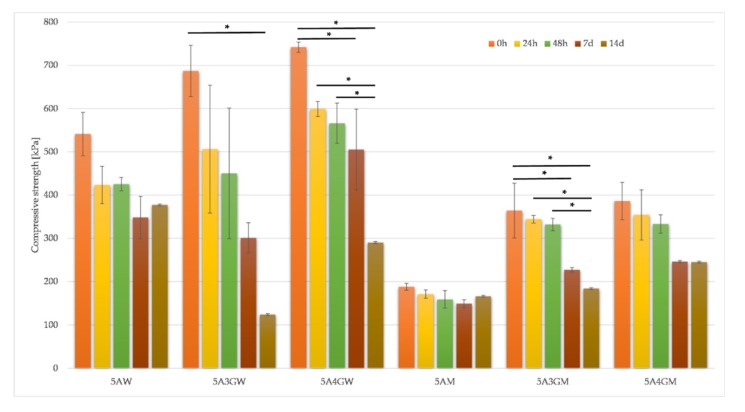
Compressive strength results for samples dissolved in water (W) and full culture medium (M) with different content of gelatine evaluated after 24 h, 48 h, 7 days, and 14 days of incubation (* = *p* < 0.05, results statistically significant).

Target applications of the printed hydrogels determine requirements for their mechanical properties. The conducted uniaxial compression tests let specify compressive strength of test samples. The results are shown in Figure 4.

The results of the hydrogels’ compressive strength after degradation showed that in the case of samples prepared using water as a solvent the compressive strength after 14 days of incubation drops more than twice for the 5A4GW sample and more than five times for the 5A3GW sample in relation to the initial value. In the case of full culture medium solvent, the decrease in compressive strength for all samples does not exceed 50% for each incubation period. In addition, for the 5AM hydrogel, the smallest changes in compressive strength values were obtained, which indicates its highest stability. For 5AW and 5AM, an increase in compressive strength after 14 days of incubation was observed compared to the 7-day period, which may have resulted from the material preparation step.

### 3.4. Results of Fatigue Measurement

**Figure 5:**
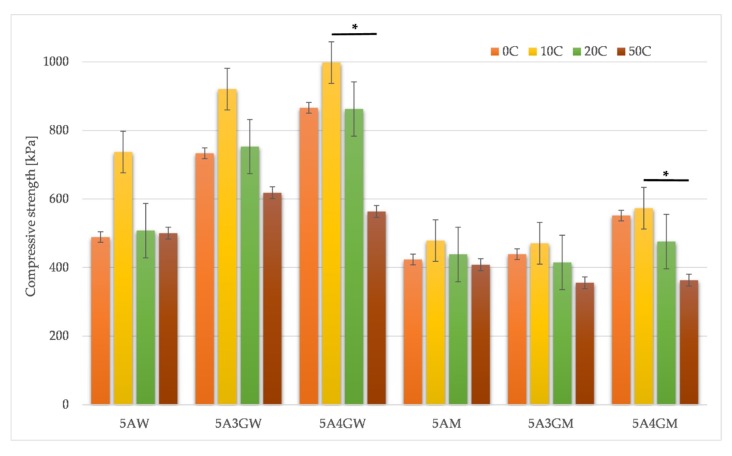
Compressive strength results for samples dissolved in water (W) and full culture medium (M) with different content of gelatine after 0 (0 C, reference samples), 10 (10 C), 20 (20 C), and 50 (50 C) cycles of loading and unloading (* = *p* < 0.05, results statistically significant).

Hydrogels were subjected to a fatigue test to determine their resistance to cyclic loads. The results are shown in Figure 5.

For all hydrogels, during 10 cycles of hydrogel loading and unloading, an increase in compressive strength was obtained. With this number of cycles, deformation of the hydrogels can result in the removal of pores and thereby the compressive strength increased. However, after 20 and 50 load cycles of the samples, the compressive strength decreases for all materials. In relation to the original value, the largest differences were observed for the samples 5A4GM and 5A4GW. Samples of alginate hydrogels without gelatine show the greatest stability and resistance to fatigue at 20 and 50 cycles. The use of various solvents affects the values of compressive strength, as shown in earlier results. For the culture medium as a solvent in the case of the 5A4GM sample, the initial compressive strength is much higher than for the 5AM sample, however, after 50 load cycles the difference disappears. Comparing samples with the same gelatine content (5A4GM and 5A4GW) both for the initial measurement and after 50 loading cycles, higher compressive strength values were obtained in the case of hydrogels prepared using water.

### 3.5. Results of Hydrogels’ Swelling Ratio

**Figure 6:**
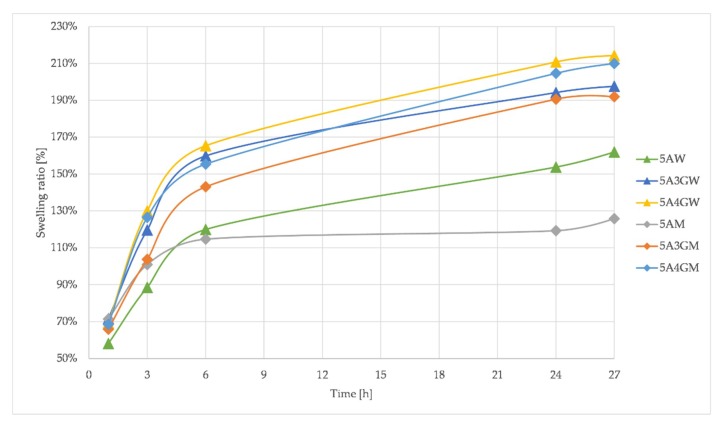
Swelling ratio for samples dissolved in water (W) and full culture medium (M) with different content of gelatine measured after 1 h, 3 h, 6 h, 24 h, and 27 h.

The ability to absorb liquids is an important feature of hydrogels used in tissue engineering. It involves the absorption of body fluids, as well as the penetration and transport of nutrients and metabolic products. Figure 6 presents the results of measurements of the swelling ratio of the tested hydrogels.

The addition of gelatine increases the swelling ratio for both types of solvents. The type of solvent also changes the swelling ratio—higher values are observed for hydrogels prepared using deionized water. This confirms the results of the carried out degradation tests—higher ability of the material to absorb aqueous solutions correlates with the increased susceptibility to the hydrolysis reaction. Alginate hydrogels with the addition of gelatine reach equilibrium after 24 h. For hydrogels prepared without gelatine after 24 h, there is a further increase in the absorbed mass of PBS buffer.

### 3.6. Results of the Cells Viability/Cytotoxicity Test after Direct Contact with Hydrogels Sample

**Figure 7:**
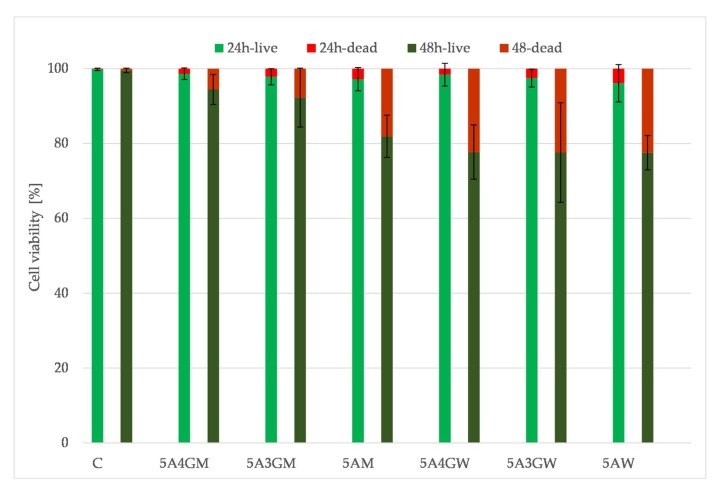
Endothelial cells (EA.hy 926 line) viability after direct contact with samples dissolved in water (W) and full culture medium (M) with different content of gelatine after 24 and 48 h of incubation.

Figure 7 shows the results of the direct viability/cytotoxicity test of the hydrogels in direct contact with cells. The determined cells’ viability decreases with the prolonged incubation period of the hydrogels. However, they do not cause a cytotoxic effect (according to the PN-EN ISO 10993-5 guidelines)—the proportion of dead cells in their total amount does not exceed 30%. The decreasing viability of cells is associated with the decreasing amount observed on the surface, which may result from their penetration into the interior of the tested samples at various depths. The highest viability after both incubation periods was obtained for the 5A4GM hydrogel. The use of full culture medium as a solvent for the hydrogels preparation stage ensures higher cell viability and their proliferative capacity. The introduction of gelatine also increases cell viability, but it also depends on the solvent used. In the case of a culture medium application, this tendency is stronger, whereas in case of water this trend is maintained at an incubation period of 24 h. After 48 h, the viability of cells seeded on hydrogels prepared with water remains at the same level.

**Figure 8:**
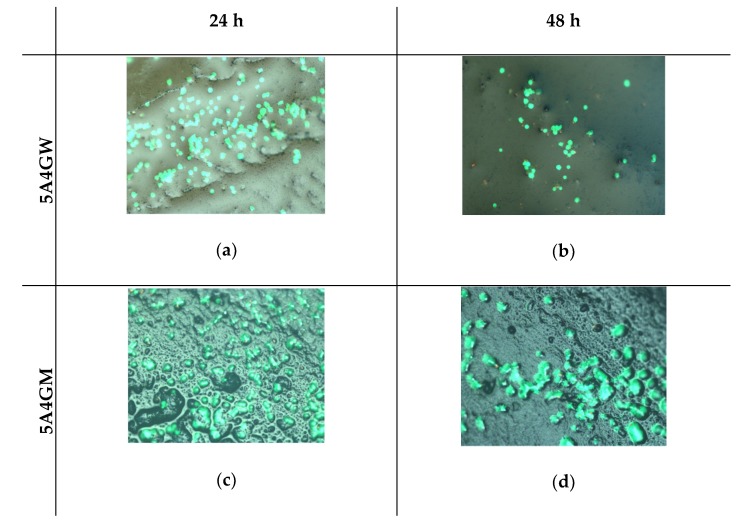
Endothelial cells (EA.hy 926 line) cytotoxicity after direct contact with 5A4GW and 5A4GM samples after 24 (**a**,**c**) and 48 h (**b**,**d**) of incubation (live cells are marked green, dead in red). Magnification 100x.

The ability of cells to adhere to the surface of hydrogels depends on their texture and the degree of porosity. Despite of the degassing process of hydrogels before cross-linking, porosities are visible during microscopic observations. Decreasing trend in cell viability can result from the fact that cells penetrate the micropores where they have a limited ability to exchange metabolic products and become dead. That is illustrated by a decreasing trend in the number of cells after 48 h in the direct test. Figure 8 presents results of cytotoxicity assessment with microscopic images of hydrogel surface texture for samples 5A4GW and 5A4GM.

### 3.7. Results of the Cells Viability/Cytotoxicity Test after Contact with Extracts Made from Hydrogels Sample

**Figure 9:**
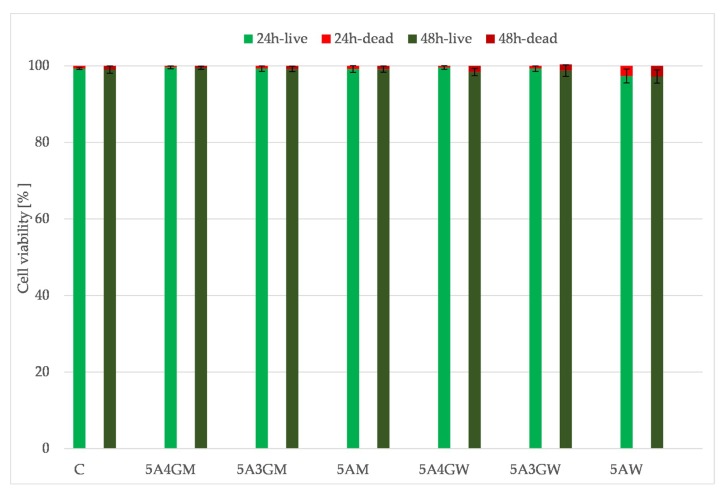
Endothelial cells (EA.hy 926 line) viability after indirect contact with samples dissolved in water (W) and full culture medium (M) with different content of gelatine after 24 and 48 h of incubation.

Figure 9 shows the results of viability assessment for an indirect test. Obtained results showed that the prepared extracts do not cause cytotoxic effects in relation to the EA.hy926 cells line (the viability does not drop below 70% both after 24 h and 48 h of incubation). The highest viability was obtained for the 5A4GM sample. 

### 3.8. Results of Extrusion Ability and Cells Survival Test after Extrusion

On the basis of previous research, one hydrogel was selected to be used as a material to prepare a bioink. Direct 3D bioprinting was carried out for the 5A4GM hydrogel, due to having good mechanical properties and the highest biocompatibility.

The temperature of the nozzle influences the cells viability. It increases with the growing temperatures, but these are not statistically significant differences. This is due to the hydrogels’ viscosity at the temperatures of 40 °C which is the lowest, and thus the lowest shear stresses are generated, what protects the cells. Analysing the influence of temperature on the hydrogel’s extrudability, the higher the temperature, the more fluid its form is, which results in printing wider paths than the diameter of the nozzle used. The obtained results indicated that the printed hydrogel paths are not uniform throughout the entire length. Furthermore, despite conduction of the degassing process of the hydrogel, the pores are visible.

## 4. Discussion

The properties of sodium alginate/gelatine hydrogels have been characterized in terms of their applicability in bioprinting and thus in tissue engineering. The work demonstrates that the properties of these materials can be modified by the use of various solvents and gelatine content. Their usefulness in printing can also be supported by introducing an extruder heating system, which was proved by obtained results.

In the case of tested dynamic viscosity of hydrogels, higher values were obtained for samples prepared with the use of water as a solvent. This tendency is preserved for materials with introduced gelatine, while in the case of alginate reference samples, a higher viscosity is achieved for those dissolved in a culture medium. This dependence is observed because medium has calcium cations, which initiate ionic crosslinking of alginate hydrogel. Calcium cations from medium diffused into the sodium alginate producing gel, the viscosity of which is higher that viscosity of alginate solution in water. In the case of hydrogels containing gelatine, viscosity dependencies tend to be reversed—for water (as a solvent) the viscosities are higher than for medium. The reason for this may be a phenomenon of interactions between sodium alginate and gelatine. In the case of water, there are no disturbed electrostatic interactions between negatively charged groups of sodium alginate and positively charged groups of gelatine. In the case of DMEM medium, it contains salts that partially separate the alginate from gelatine and the interactions between these components are weaker, which has the effect of a less intense increase in viscosity. The influence of the addition of gelatine on the viscosity increase confirms results presented by Colosi et al. [42]—after the introduction of gelatine, the viscosity increases three-fold compared to the alginate hydrogel. Increased viscosity requires the use of higher pressures to extrude the hydrogels, but prevents the automatic outflow of the gel from the nozzle and ensures greater accuracy of the printed structures.

**Figure 10:**
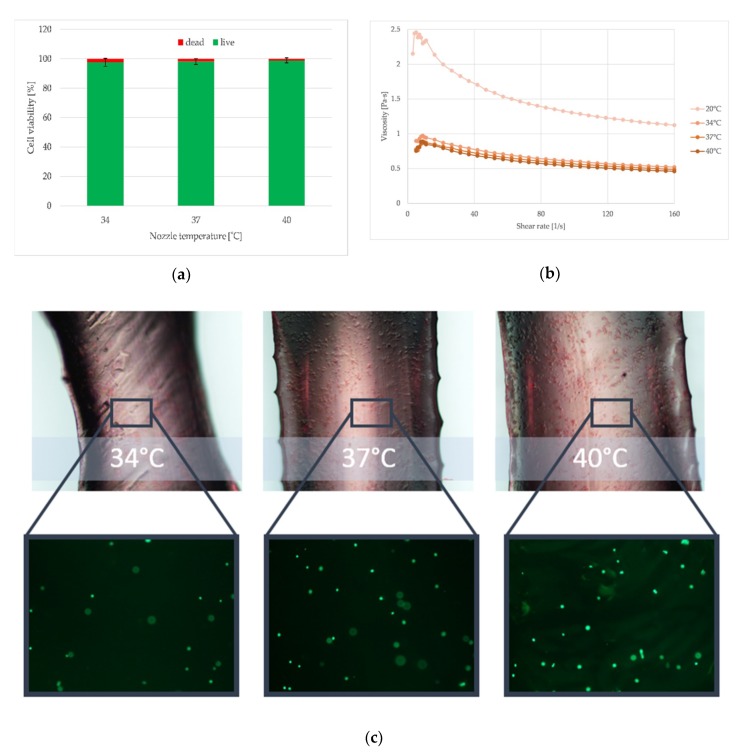
Influence of different temperatures of extrusion (34, 37, and 40 °C) on the endothelial cells (EA.hy 926 line) viability (**a**), viscosity (**b**), and path width (**c**) of the 5A4GM hydrogel. Photomicroscopy, 100× magnification, fluorescence microscopy, 50× magnification.

Apart from the concentration of sodium alginate and gelatine and the type of solvent, the rheological properties of hydrogels are also affected by their temperature. As our research showed (Figure 10), the higher it is, the lower is the viscosity of the material being tested. The introduction of the nozzle heating allowed us to control the temperature of the extruded hydrogel. In terms of the use of hydrogels in bioprinting for tissue engineering needs, this is important due to the fact that it makes it possible to use materials with higher content of various components (as e.g., attractants or cells’ growth factors) and thus also with higher viscosity. Their extrusion becomes possible by changing the temperature. Additionally, what is very important, heating hydrogels to 34, 37, and 40 °C does not significantly affect cell survival. Furthermore, when the nozzle was heated to 40 °C, the highest cell viability was obtained. This is due to the fact that at this temperature the viscosity of the tested hydrogel is the lowest and the lowest shear stress is generated. Nevertheless, this temperature must be selected so as not to cause thermal destruction of the cells. For printouts with a nozzle temperature of 34 °C, cell viability decreased, what is related to the increasing viscosity.

Compressive strength increases with the increasing proportion of gelatine, as already confirmed in previous studies [44,47]. This property was observed both for low (1, 2, 4% sodium alginate and gelatine up to 4%) and for higher concentrations (5% sodium alginate with gelatine up to 10%). Our results confirmed this trend for both types of solvents. A further increase in compressive strength is possible by increasing the concentration of sodium alginate and gelatine, but this results in a simultaneous increase in viscosity, which limits the printability and exposes the cells suspended in the hydrogel to high shear stress. In addition to the effect of the concentration of sodium alginate and gelatine, the influence on the compressive strength values depending on the solvent used was also demonstrated. The use of water as a solvent improves mechanical properties, but at the same time increases the viscosity of the hydrogel. At the sample preparation stage, the same crosslinking times were used for the both types of solvents. Because the crosslinking process occurs slower for the culture medium than for the water (due to the composition of the medium and therefore the formation of separating salts), the samples may have different levels of crosslinking. This is manifested in a lower level of compressive strength values for culture medium than for the water (for these samples we have a higher level of crosslinking in the entire volume). The addition of gelatine influences the values of compressive strength. The reason for this may be a phenomenon of interactions between sodium alginate and gelatine (what was mentioned above). According to Xing et al. [48] higher electrostatic interactions cause higher density of crosslinking and in consequence improve mechanical strength.

The analysis of the results from the conducted tests confirms the gradual degradation of the tested hydrogels. The choice of solvent in the hydrogel preparation step has a significant impact on the speed of this process. Samples made using water degrade faster, as evidenced by a higher degradation index and a greater drop in compressive strength. The degradation rate also depends on the hydrogel composition. With the increasing concentration of gelatine, degradation processes are accelerated, which has already been noticed in the research conducted by Rosellini’s et al. [49]. This may be due to the fact that at 37 °C gelatine is a sol and in such a liquefied form it can be more easily washed out from the hydrogel. Conducting the test using PBS accelerates degradation probably because calcium ions of Ca^2+^ are exchanged in the structure of crosslinked alginate to sodium Na^+^ ions from PBS buffer [44], especially in the case of water as the solvent. The occurrence of the exchange reaction is accelerated by the growing temperature. The lack of calcium ions reverses the crosslinking reaction, which reduces the mechanical properties and causes loss of mass. This was confirmed by studies carried out by the Giuseppe et al. [44], whose research showed a significant decrease in mechanical properties as a result of incubation in PBS at 37 °C. Simultaneously, incubation in the same solution, but at a much lower temperature of 3 °C, slows down the loss of mechanical properties, which results from the slowing of the calcium ion exchange. In the case of DMEM culture medium, process of the exchange of Ca^2+^ ions with Na+ ions may take place more slowly due to the fact that at the stage of hydrogel’s preparation; salts, which are formed with ions from DMEM, are chemically more stable than calcium salts, and the exchange of ions is more difficult. That is why degradation rate for sodium alginate/gelatine hydrogels dissolved in water is higher than for those dissolved in culture medium (Figure 3), although some mechanical properties (e.g., compressive strength presented in Figure 4) are higher for water solvent. It can be seen, however, that the hydrogels dissolved in the medium are more stable over time. In the case of the 5A4GM sample the increasing rate of degradation and falling mechanical properties up to day 7 may result from the ongoing calcium ion exchange reactions. The maintenance of mechanical properties after 14 days at the same level in relation to the results for 7 days may be related to the depletion of sodium ions after this time and stopping the exchange reaction [44].

In the case of hydrogels prepared using water, the swelling coefficient reaches higher values. This may explain the higher rate of degradation processes occurring, because the alginate hydrogels undergo hydrolytic degradation in aqueous solutions [50]. Thus, the higher water absorption capacity will be responsible for providing it in greater amounts to the internal structure of the hydrogels and, as a result, accelerates the occurrence of these processes, which is consistent with the fact that degradation is progressing faster for these materials. The ability to absorb liquids also increases by the addition of gelatine—the higher its content, the higher the swelling ratio for both types of solvent. The determined values of the swelling ratio did not exceed 230% for all the materials. For comparison, in the research conducted by Pan et al. [40], a hydrogel consisting of sodium alginate (0.04 g/mL) and gelatine (0.2 g/mL) crosslinked with the use of calcium chloride, had an ability to absorb water at the level of 700%. Apart from the selection of the composition and solvent, this is also influenced by the crosslinking method, which was confirmed by Bigi et al. [51]. Their research had shown that the higher the concentration of the crosslinking agent, the lower is the swelling ratio.

The results of conducted fatigue tests of hydrogels confirmed the influence of cyclic loads on the compressive strength of hydrogels. An interesting result is the fact that the compressive strength increases after 10 loading and unloading cycles of the sample. It is possible that in this range air bubbles are removed from the hydrogel structure, which leads to homogenization of the material and increases the final value of compressive strength. Another reason for compressive strength increase after 10 load cycles is the influence of the swelling ratio. In the work carried out by Bandyopadhyay et al. [46], swelling coefficient was examined on printed structures made of sodium alginate/gelatine hydrogels. It has been shown that they have the ability to absorb water until reaching equilibrium (the maximum degree of water saturation, as demonstrated by the stabilization of the sample mass), and the determined swelling ratio was over 25% after 27 h of incubation. Performing 10 load cycles may cause absorption of the PBS buffer in which the samples are immersed, and thus the compressive strength value is higher. The increase in the number of cycles up to 20 and 50 already affects the materials. Cyclic deformation of the material leads to the breaking of bonds inside the chains forming the structure of the hydrogels. As a result of such fatigue, the compressive strength of the material decreases.

The evaluation of cytotoxicity of materials was carried out in two ways: through direct contact with hydrogels and in the test with extracts prepared from these materials (indirect contact) in accordance with guidelines given in PN-EN ISO 10993-5. The use of various solvents indirectly affects the cells viability and their ability to proliferate. Hydrogels prepared using a culture medium provide a better environment for cell growth. As shown in the literature, the cells’ ability to adhere to the tested hydrogel samples depends on the introduction of gelatine [52] and topography of the surface. The cells selectively choose areas on the test samples for growth. Our results confirm this also. Hydrogels prepared using DMEM culture medium were characterized by a higher proportion of micropores. Growing cells adapt to the shape of the surface, hence their unnatural shapes in the microscopic image. However, when the cells reach the pores with dimensions that prevent their further growth, they undergo necrosis, due to the unsuitable environment for growth, the lack of nutrients from the medium, and the impossibility of removing waste products. The tests carried out with the use of hydrogel extracts confirmed that they are a non-toxic material regardless the type of solvent and chemical composition. A high level of open porosity with appropriate pore sizes may allow cells to migrate, access the medium and multiply. Bearing in mind the obtained results, even better results in the field of cell proliferation (and thus very rapid creation of tissue structures) can be obtained for the developed hydrogels by deliberately introducing air bubbles of defined sizes (open-pored structure).

Prior to performing the direct 3D bioprinting test, the effect of the preparation step of the bioink (made of chosen 5A4GM hydrogel) on the viability of the cells was evaluated. The obtained results showed 100% viability, which confirmed the correctness of the procedure and made it possible to assess the impact of the printing process on the cells contained in the bioink.

As demonstrated in this work, the properties of hydrogels sodium alginate/gelatine can be modified in several ways in order to prepare biocompatible bioink having good extrudability in additive manufacturing process. In the field of research on the effect of the solvent used, a few works can be found. Although studies of other research teams have already indicated the effect of PBS buffer concentration on mechanical properties, biological response and printability [45], none of them includes testing for a culture medium as a solvent.

## 5. Conclusions

In this paper, it was shown that the use of deionized water as a solvent for the production of sodium alginate/gelatine hydrogels accelerates the degradation processes, as evidenced by a higher degradation index and a significant decrease in compressive strength. Hydrogels dissolved in water have a higher water absorption capacity which is improved by the addition of gelatine. The use of a culture medium as a solvent increases the viability of cells seeded both directly on hydrogels and in contact with extracts. At the same time, the medium causes a greater hydrogel porosity.

Despite applied solvent, increasing concentration of gelatine has been shown to increase cell viability. On the basis of rheological tests, it has been shown that the introduction of gelatine to sodium alginate increases the viscosity of the hydrogel, which additionally depends on the temperature of the material being tested (temperature increase decreases the viscosity of the hydrogel). Heating the nozzle to 40 °C during the bioprinting process reduces the viscosity of the hydrogel, thereby reducing the generated shear stress and increasing the survival rate of the cells after printing.

Bearing in mind the obtained results, the bioink extrusion parameters can be influenced by hydrogel composition and temperature during the printing process in order to produce highly defined 3D biostructures.

## Figures and Tables

**Table 1:** Applied signatures of the tested materials, the concentration of sodium alginate and gelatine and kind of applied solvents.

Signature	Concentration		Solvent Short Name
	Sodium Alginate (% *w*/*v*)	Gelatine (% *w*/*v*)	
5A W	5	-	W = water ^1^
5A3G W	5	3	W
5A4G W	5	4	W
5A M	5	-	M = medium ^2^
5A3G M	5	3	M
5A4G M	5	4	M

^1^ Water = deionized water. ^2^ Medium = DMEM + 10%FBS + 1%P/S (full culture medium).
